# Supplementary material for: Spongin as a Unique 3D Template for the Development of Functional Iron-Based Composites Using Biomimetic Approach In Vitro
Source: Mar Drugs. 2023 Aug 22;21(9):460. doi: 10.3390/md21090460 (PMC10532518; doi:10.3390/md21090460)
Supplement: Supplementary file 1 [file marinedrugs-21-00460-s001.zip › marinedrugs-2527227-supplementary/Supplementary.docx]

Supplementary information for article

Spongin as unique 3D template for development of Iron-based composites using biomimetic approach in vitro

Anita Kubiak ^1,2*^[
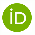
](https://orcid.org/0000-0002-3310-3239),Martyna Pajewska-Szmyt^2^[
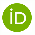
](https://orcid.org/0000-0003-2308-2750), Martyna Kotula^1,2^[
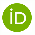
](https://orcid.org/0000-0002-0231-130X), Bartosz Leśniewski^1,2^[
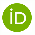
](https://orcid.org/0000-0003-2178-9648), AlonaVoronkina ^3,4^[
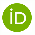
](https://orcid.org/0000-0003-2750-0884), Parvaneh Rahimi ^3^[
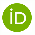
](https://orcid.org/0000-0002-8858-5407), Sedigheh Falahi ^3^, Korbinian Heimler^5^[
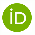
](https://orcid.org/0000-0003-3385-1808),Anika Rogoll^5^[
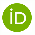
](https://orcid.org/0000-0002-4144-2353), Carla Vogt^5^[
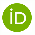
](https://orcid.org/0000-0002-6711-0103), Alexander Ereskovsky^6^[
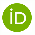
](https://orcid.org/0000-0003-1079-7204), Paul Simon^7^, Enrico Langer^8^, Armin Springer ^9,10^, Maik Förste^11^, Alexandros Charitos^11^,Yvonne Joseph^3^[
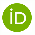
](https://orcid.org/0000-0003-0697-9646), Teofil Jesionowski^12^[
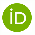
](https://orcid.org/0000-0002-7808-8060),Hermann Ehrlich^2,12*^[
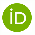
](https://orcid.org/0000-0003-4951-3555)

^1^ Faculty of Chemistry, Adam Mickiewicz University, Uniwersytetu Poznańskiego 8, 61-614 Poznan, Poland; markot6@amu.edu.pl (M.K.); barles5@amu.edu.pl (B.L.);

^2^ Center of Advanced Technology, Adam Mickiewicz University, Uniwersytetu Poznańskiego 10, 61-614 Poznan, Poland; mpszmyt@amu.edu.pl (M. P-Sz.);

^3^ Institute of Electronic and Sensor Materials, TU Bergakademie Freiberg, Gustav-Zeuner-Str. 3, 09599 Freiberg, Germany; voronkina@vnmu.edu.ua (A.V.), parvaneh.rahimi@esm.tu-freiberg.de (P.R.); sedigheh.falahi@doctorand.tu-freiberg.de (S.F); yvonne. joseph@esm.tu-freiberg.de (Y.J.);

^4^ Department of Pharmacy, National Pirogov Memorial Medical University, Vinnytsya, Pyrogov str. 56, 21018 Vinnytsia, Ukraine;

^5^ Institute of Analytical Chemistry, TU Bergakademie Freiberg, Leipziger Str. 29, 09599 Freiberg, Germany; korbinian.heimler@chemie.tu-freiberg.de (K.H.); anika.rogoll@chemie.tu-freiberg.de (A.R.); carla.vogt@chemie.tu-freiberg.de (C.V.);

^6^ IMBE, CNRS, IRD, Aix Marseille University , Station Marine d’Endoume, Rue de la Batterie des Lions, 13007 Marseille, France; alexander.ereskovsky@imbe.fr;

^7^ Max Planck Institute for Chemical Physics of Solids, Nöthnitzer Str. 40, Dresden 01187, Germany; simon@cpfs.mpg.de;

^8^ Institute of Semiconductors and Microsystems, TU Dresden, Nöthnitzer Str. 64, 01187 Dresden, Germany; enrico.langer@tu-dresden.de;

^9^ Department Life, Light & Matter, University of Rostock, Albert-Einstein-Str. 25, 18059 Rostock, Germany

^10^ Medical Biology and Electron Microscopy Centre, Rostock University Medical Center, Strempelstr. 14, 18057 Rostock, Germany,

armin.springer@med.uni-rostock.de;

^11^ Institute for Nonferrous Metallurgy and Purest Materials (INEMET), TU Bergakademie Freiberg, Leipziger Str. 34, D-09599 Freiberg, Germany, maik.foerste@inemet.tu-freiberg.de (M. F.);alexandros.charitos@inemet.tu-freiberg.de (A.C.)

^12^ Faculty of Chemical Technology, Institute of Chemical Technology and Engineering, Poznan University of Technology, Berdychowo 4, 60965 Poznan, Poland; teofil.jesionowski@put.poznan.pl;

***** Correspondence: anikub@amu.edu.pl (A.K.); herehr@amu.edu.pl (H.E.);


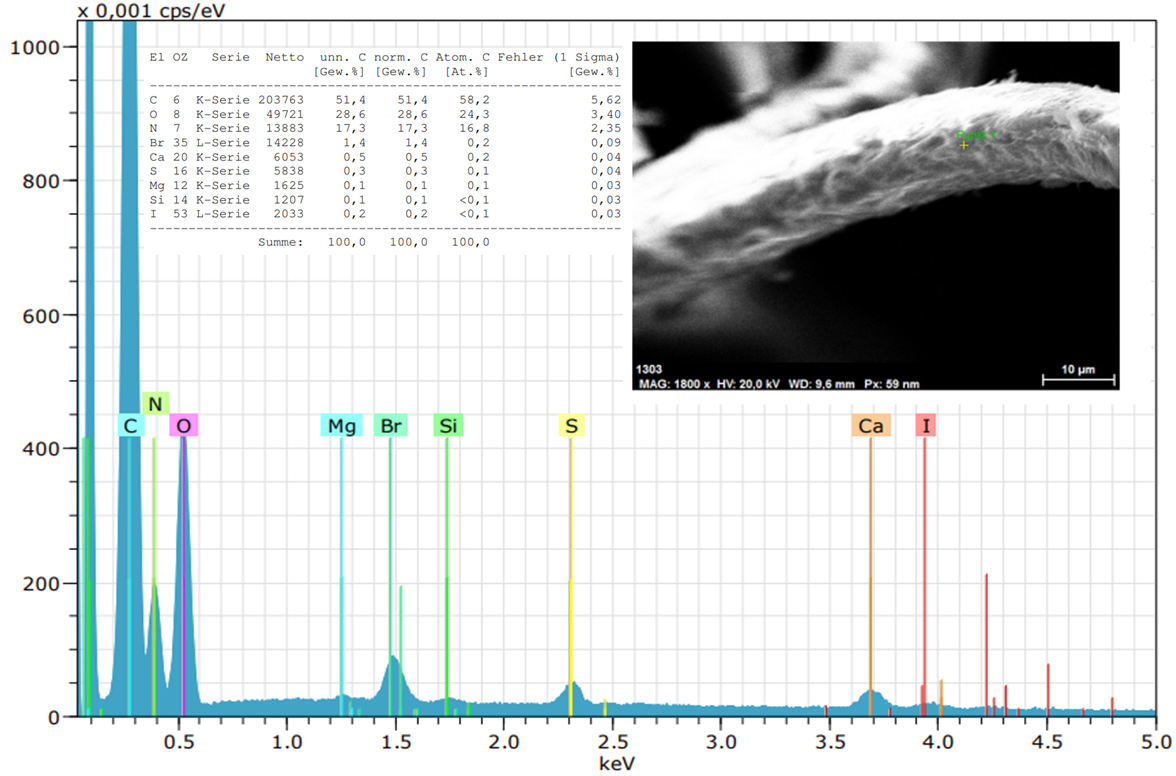


**Figure S1.** EDX measurements of pure spongin scaffold isolated from *Hippospongia communis* demosponge (control sample).


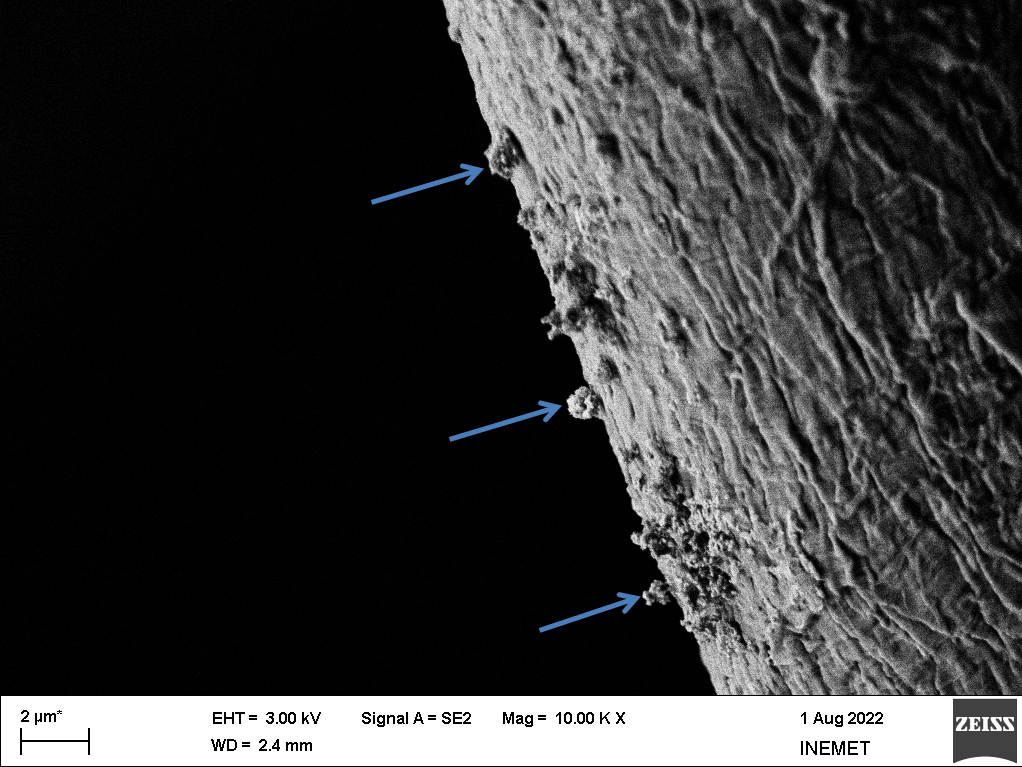


**Figure S2.** SEM image of lepidocrocite nanoparticles formed on the surface of natural spongin fibre of *H. communis*, described as rusty sponge.


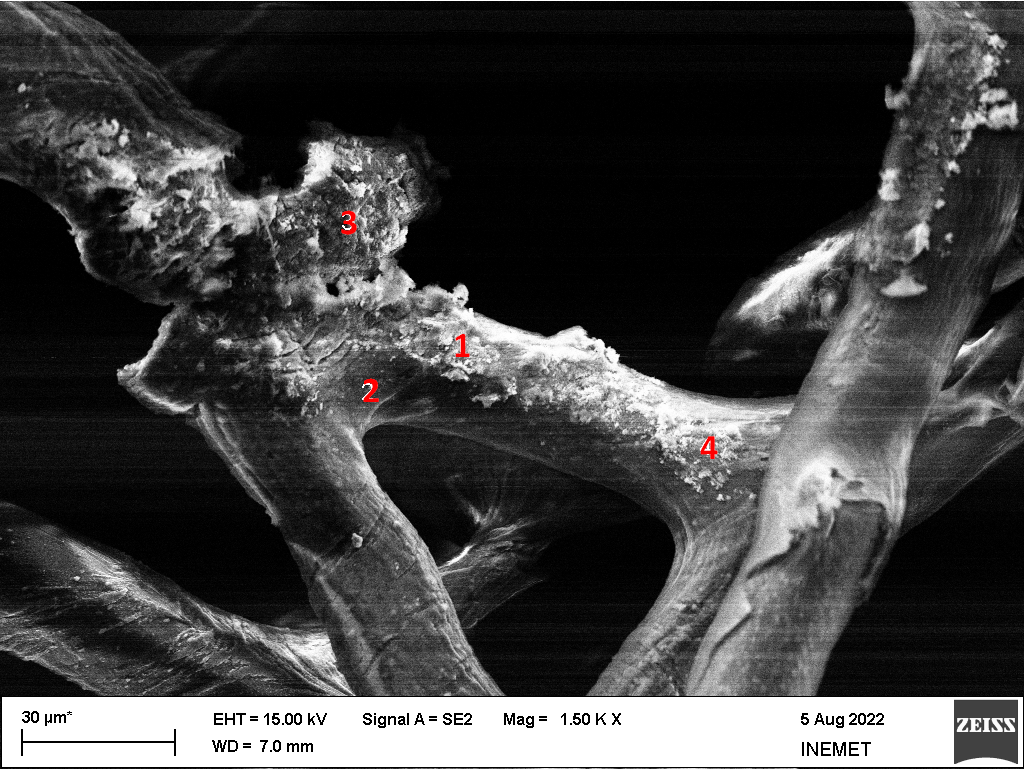


**Figure S3.**SEM image with spots of EDX measurements of the rusty natural spongin scaffold of *H.communis*, (for measurements data see Figure S4).

| 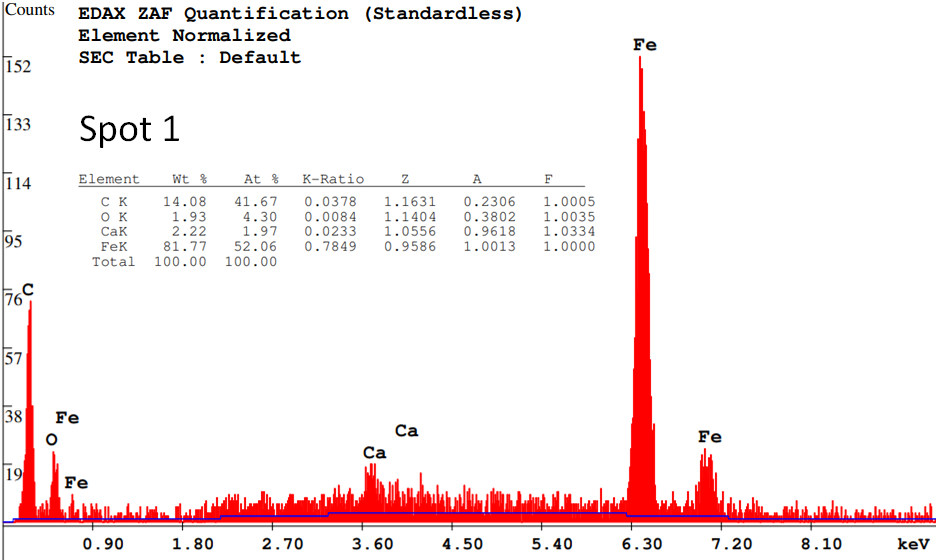  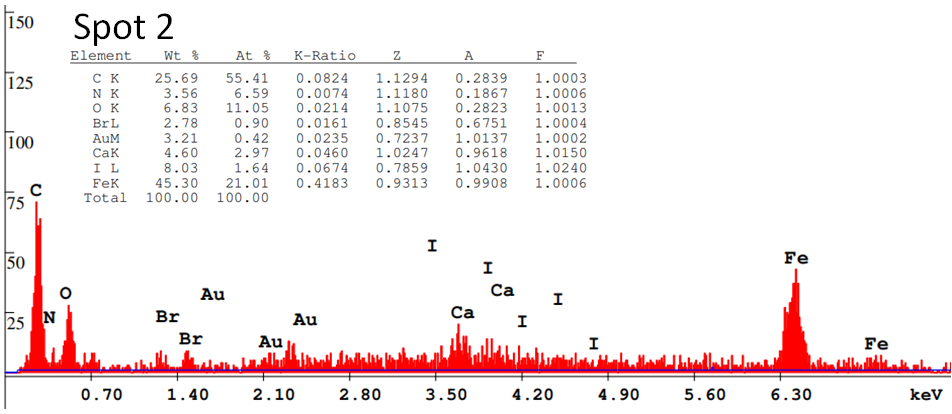  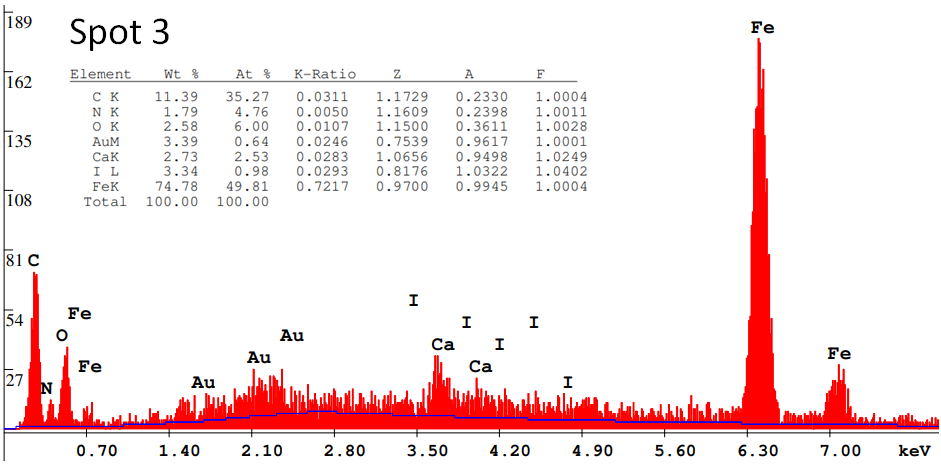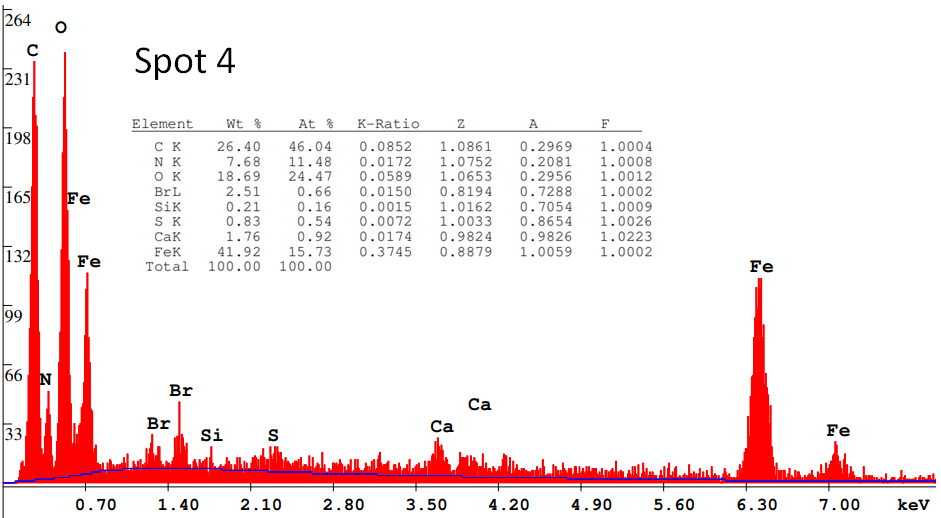 |  |
| --- | --- |

**Figure S4.** EDX measurements for the natural rusty spongin scaffold of *H.communis*, (for spots locations see Figure S3).

| 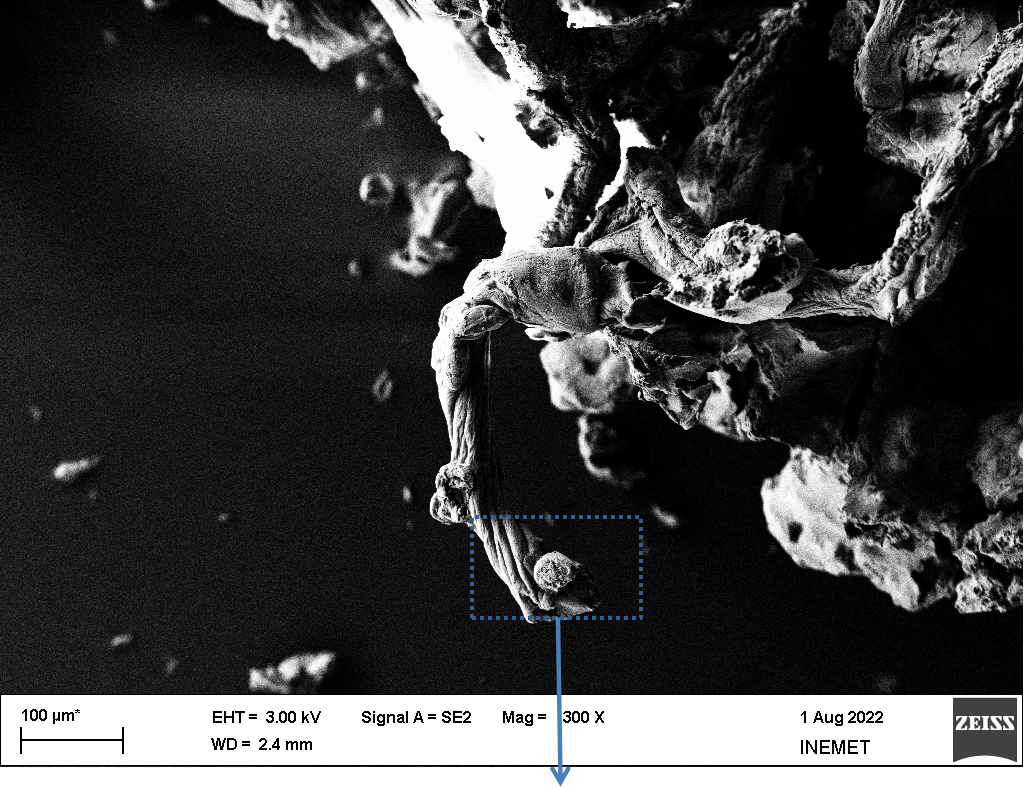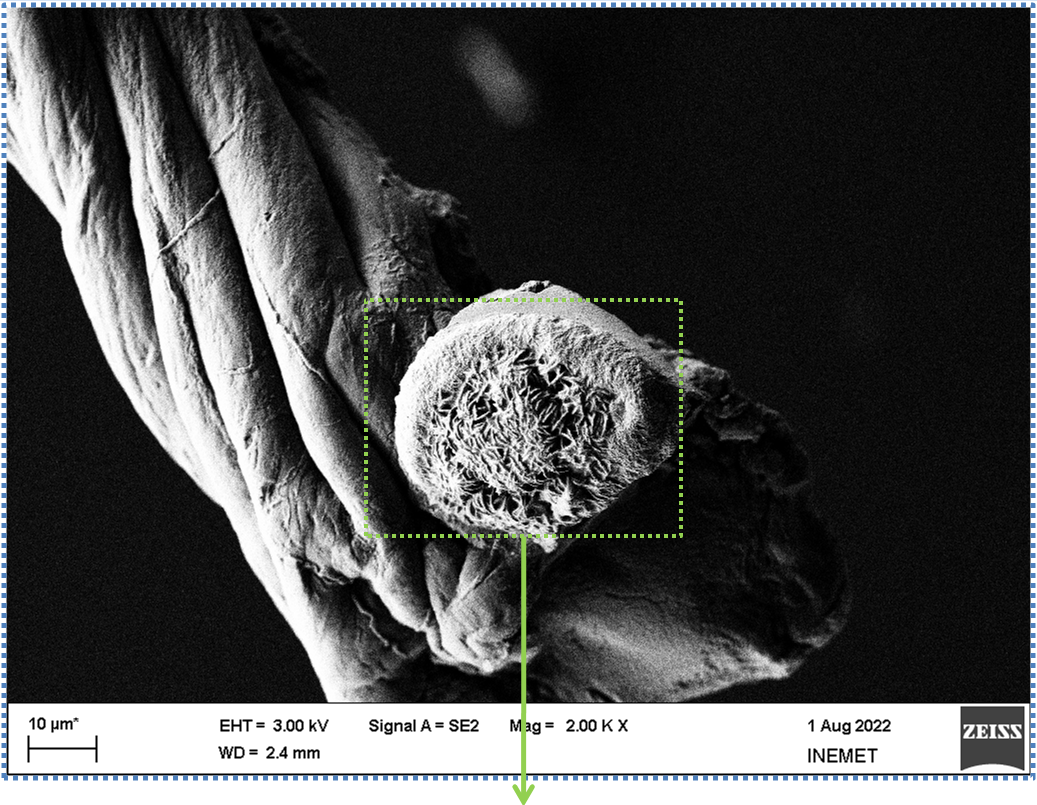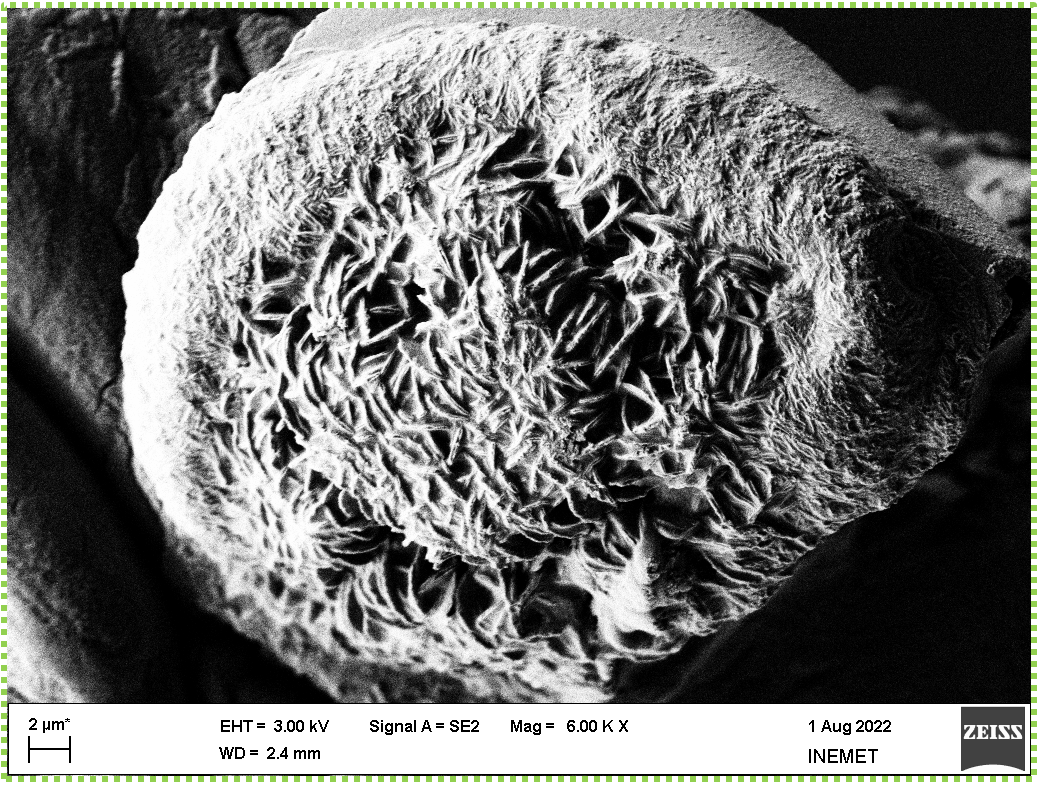 | . |
| --- | --- |

**Figure S5.** SEM images of the “Iron-Spongin after ultrasound treatment”scaffold with well defined crystals of lepidocrocite. See also Fig.S6.

**
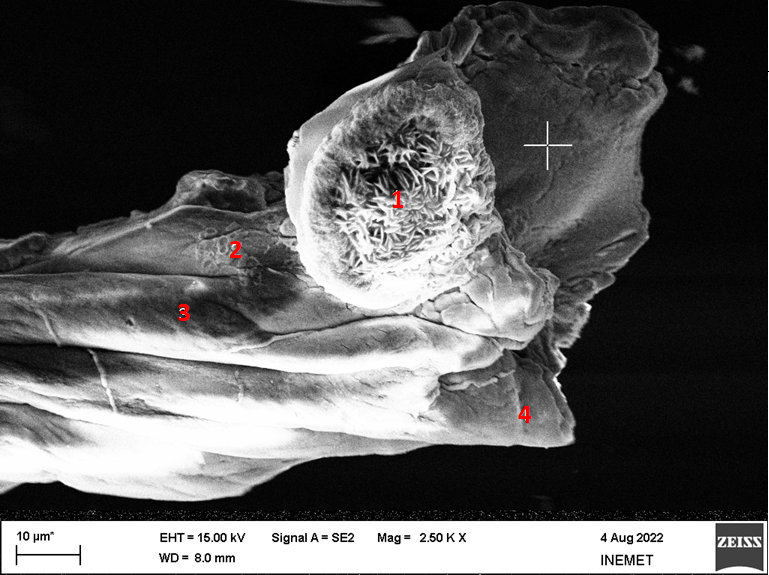
**

**Figure S6.** SEM image with spots of EDX measurements carried out on “Iron-Spongin after ultrasound treatment” sample (for measurements data see Figure S7).

| 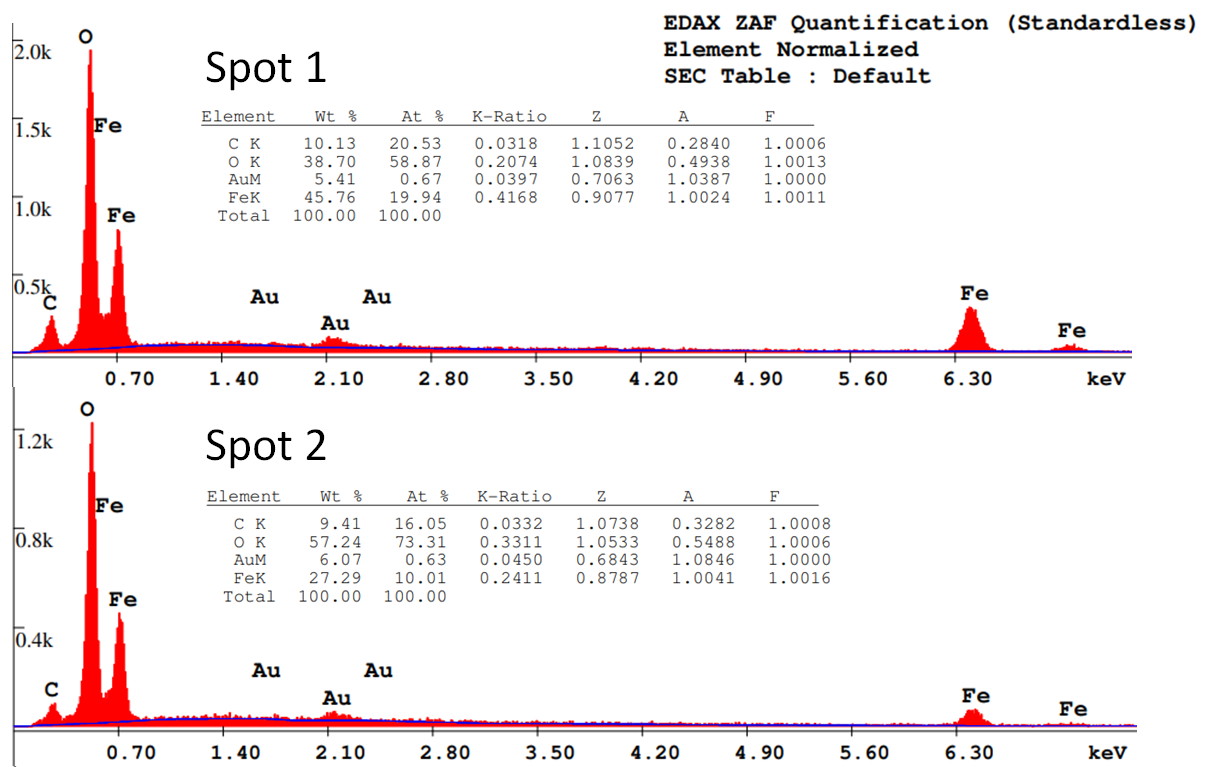  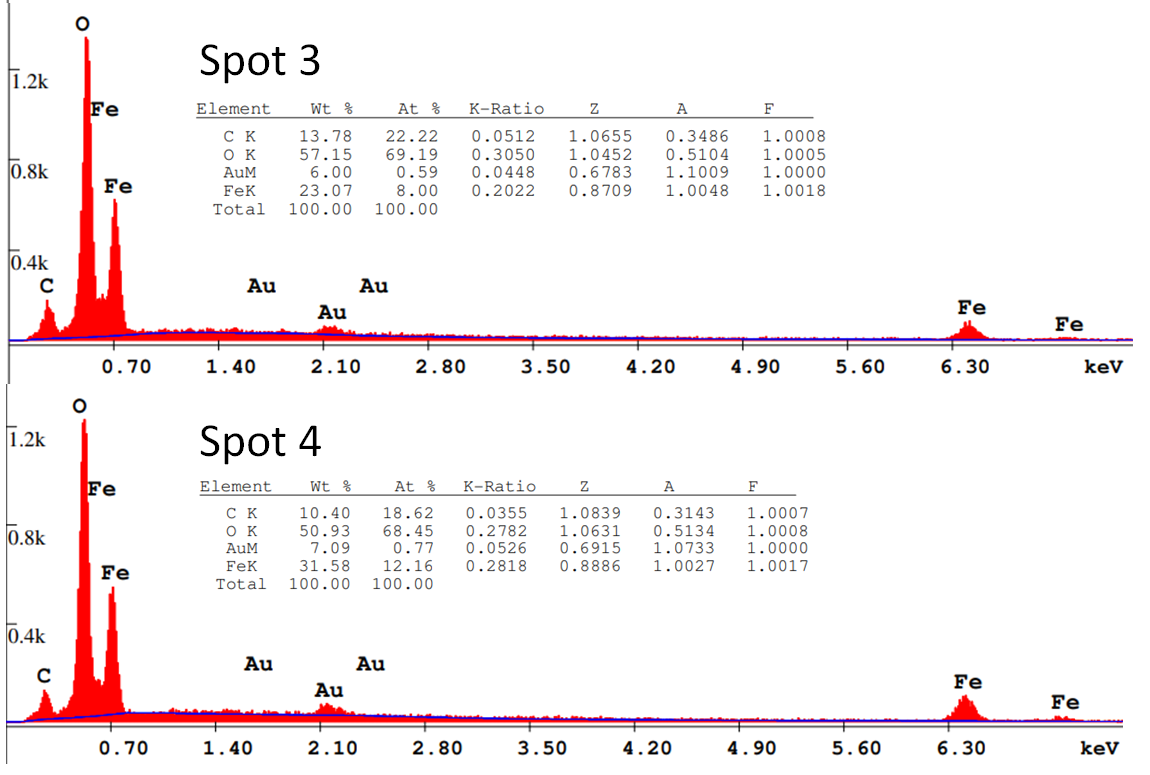 |  |
| --- | --- |

**Figure S7.** EDX measurements for the “Iron-Spongin” scaffold (for spots locations see Figure S6).

| 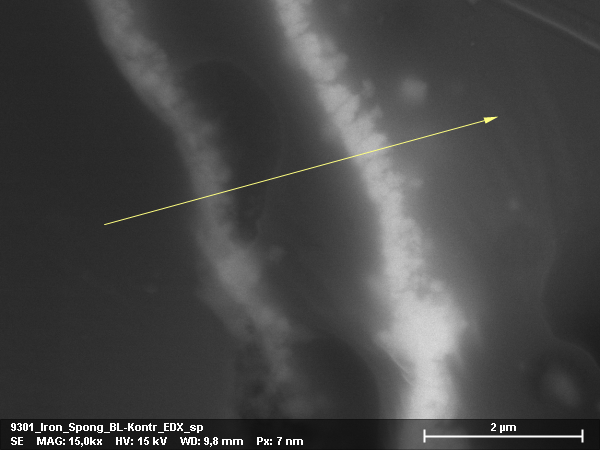 |
| --- |
| 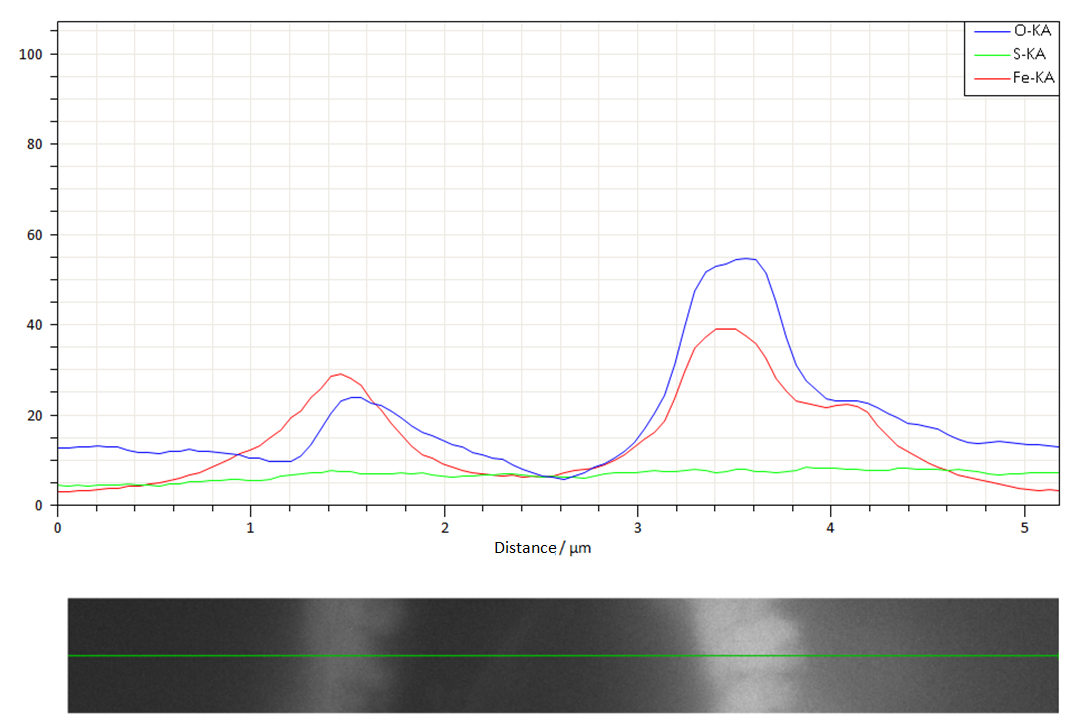 |

**Figure S8.** Quantitative EDX analysis (line scan) of a block face image cross section of a single “Iron-Spongin after ultrasound treatment” fiber. The elemental distribution of Fe, O and S along the pathway from the outer layer to the inner one: the outer layer and the inner layer contains Fe and O respectively. Noticeable are the differences in content of Fe and O in the outer and inner layer. S shows no differences within the analysed line.

**Table S1.** Wavenumbers of the bands present in the spectra of the studied samples and their assignment.

| **Spongin pure**  **sample in seawater** | **Iron-Spongin** | **Iron-Spongin**  **after ultrasound treatment** | **Vibrational**  **assignment** |
| --- | --- | --- | --- |
| 3410 | 3409 | 3415 | –OH stretching |
| 3300 | 3302 | 3290 | –NH stretching |
| 2931 | 2933 | 2933 | –CH2, –CH3 stretching |
| 1630 | 1630 | 1630 | C=O stretching |
| 1528 | 1535 | 1537 | –NH deformational |
| - | 1150 | 1150 | -OH deformational |
| 1250 | 1240 | 1240 | C–N stretching |
| 1030 | 1030 | 1030 | C–O stretching |
| - | 1021 | 1021 | Fe–OH |
| - | 742 | 740 | -OH deformational |
| - | 570 | 570 | Fe–O stretching |
| 472 | 462 | 461 | N-H stretching |


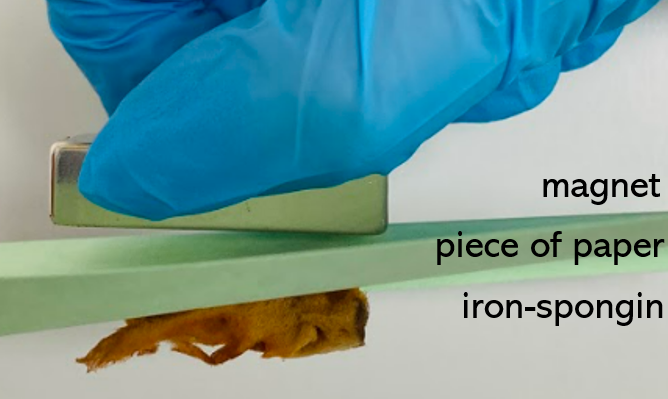


**Figure S9.** Magnetic properties of biomimetically created “Iron-Spongin after ultrasound treatment” sample.
