# Supplementary figures and images for: Spongin as a Unique 3D Template for the Development of Functional Iron-Based Composites Using Biomimetic Approach In Vitro
Source: Mar Drugs. 2023 Aug 22;21(9):460. doi: 10.3390/md21090460 (PMC10532518; doi:10.3390/md21090460)

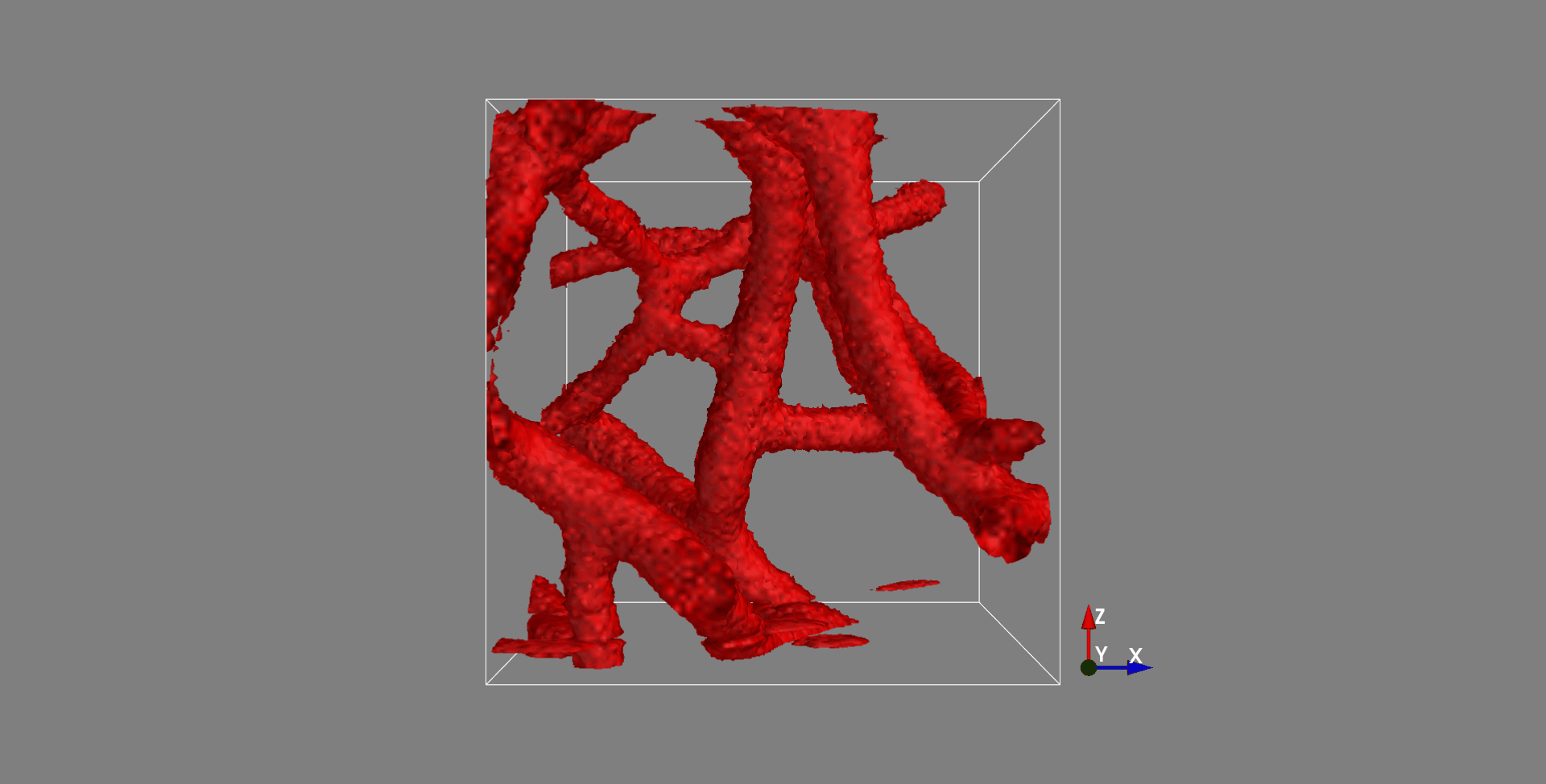

Supplement: Supplementary file 1 [file marinedrugs-21-00460-s001.zip › marinedrugs-2527227-supplementary/Supplementary Video 1 Rusty Spongin_natur_Fe-KA (1).gif]
